# Coincidence of juvenile idiopathic arthritis and type 1 diabetes: a case-based review

**DOI:** 10.1007/s00296-021-05083-z

**Published:** 2022-01-08

**Authors:** Maciej Szabłowski, Michał Andrzej Okruszko, Katarzyna Pochodowicz, Paweł Abramowicz, Jerzy Konstantynowicz, Artur Bossowski, Barbara Głowińska-Olszewska

**Affiliations:** 1 Department of Pediatrics, Endocrinology, Diabetology With Cardiology Division, Medical University of Białystok, Białystok, Poland; 2 Department of Pediatrics, Rheumatology, Immunology and Metabolic Bone Diseases, Medical University of Białystok, Białystok, Poland

**Keywords:** Autoimmune diseases, Children, Comorbidity, Diabetes mellitus type 1, Juvenile arthritis

## Abstract

The study was aimed to review a rare coexistence of type 1 diabetes (T1D) and juvenile idiopathic arthritis (JIA) regarding different clinical approaches to the management and treatment options. Medical complications of the two autoimmune disorders in children and adolescents have been evaluated, particularly in those treated with glucocorticosteroids (GCS) and insulin. A review of the literature regarding reports on concomitant T1D and JIA was conducted using resources available in Medline, Google Scholar, and Web of Science databases, with a specific focus on the combination of T1D and JIA in a pediatric population. The review was extended by our analysis of two patients treated in a single center for this comorbidity. Eligible reports of four cases were found, and including our two original records, a total of six pediatric patients (5 females) were analyzed, of which three had also other autoimmune diseases (thyroiditis, coeliac disease, autoimmune hepatitis), whereas four had been treated with a long-term GCS, and two were receiving biological therapy (etanercept or adalimumab). Only one of them had good metabolic control of diabetes. Diabetes in childhood may coexist with other autoimmune diseases, including rheumatologic conditions. Hyperglycemia can worsen JIA therapy by induction and maintaining inflammation. Using modern diabetes technologies (like personal insulin pumps and continuous glucose monitoring) helps to minimize the deteriorating effect of JIA exacerbations and the rheumatoid treatment on metabolic control of diabetes.

## Introduction

Diabetes is regarded as a global health problem in the twenty-first century. The incidence of type 1 diabetes (T1D) has increased by 3.4% during recent decades, and the continuing rise is predicted, which confers a severe public health burden worldwide [1–4]. Some commonly observed comorbidities of T1D, like Hashimoto thyroiditis and coeliac disease, are well-known, and proper screening programs at the diagnosis of diabetes are established. Nevertheless, other conditions like juvenile idiopathic arthritis (JIA) and Addison’s disease remain less explored, so clinicians involved in diabetes care might also expect some revisited algorithms or screening tools concerning the comorbidity.

A cluster of multiple diseases in the same person implicates several clinical, social, and therapeutic issues, often leading to complications and challenging treatment decisions. The problem may emerge because of different symptoms, of which co-occurrence obscures the clinical picture of individual disease entities, making it difficult to diagnose due to polypharmacy. This manifests itself concerning autoimmune diseases in the treatment of which pleiotropic and multitargeted drugs are used. An example of such a disease is JIA. The literature shows that patients incurring this disease are at higher risk of T1D. Some research suggests that it is associated with the mutation in the PTPN22 gene encoding an enzyme inhibiting the T-cell activation pathway [5, 6]. Both the rheumatologic condition per se and a long-lasting treatment burden may significantly negatively impact T1D. Inflammation as a core element of JIA and glucocorticosteroids (GCS) use exacerbate glycemia fluctuations making it challenging to determine the insulin doses and keep them constant. A cumulative effect of those factors disturbs the diabetes metabolic control [7].

JIA and T1D are also significant public health issues in pediatric populations worldwide; two chronic diseases with the early onset in childhood, resulting in short-term and late, life-long consequences and complications, with a powerful impact on health status in adulthood. Therefore, we intended to review the contemporary therapeutic approach to these diseases and point out specific difficulties in the course of treatment, based on two cases of patients with comorbid JIA and T1D from our center, extended by the analysis of another case series reported in commonly used databases.

## Search strategy

The literature search in the electronic databases: Medline (via PubMed), Web of Science, and Google Scholar was conducted in March 2021. We used the entry keywords “Arthritis, Juvenile” AND “Diabetes Mellitus, Type 1” using MeSH thesaurus on PubMed. When searching Google Scholar and Web of Science databases, we were using "Juvenile idiopathic arthritis" AND "diabetes mellitus type 1" AND "case study" OR "case report" terms. We included the studies published from 2010 until the day of the search, published in English language and concerning pediatric patients diagnosed with both the T1D (diagnosis based on International Society for Pediatric and Adolescent Diabetes (ISPAD) [8]) and JIA (meeting the International League of Associations for Rheumatology, ILAR) classification criteria [9]). We reviewed the abstracts of the relevant studies and subsequently retrieved the appropriate articles. We also scrutinized the reference lists of the included studies to identify additional references. Four case studies that met the adopted criteria were found [10–13]. Furthermore, we reported the history of two children treated in an academic tertiary care hospital; medical records, direct examination, and clinical assessment were used. The data and summary of clinical and laboratory results of all six eligible cases are shown in Table 1.

**Table 1:** The coincidence of JIA and type 1 diabetes in the pediatric population: summary of published case studies

Study	Sex	Age at JIA diagnosis	JIA subtype (based on ILAR criteria)	Number of affected joints at JIA diagnosis	Drugs used in the course of JIA treatment	HbA1c during studied period	Daily requirement of exogenous insulin (if available)	Timing of T1D and JIA clinical manifestation	Other autoimmune diseases
Case 1	Female	1 year 8 months	RF-negative polyarthritis	7	NSAIDs, prednisone, methotrexate,	10.63% (at diagnosis), 7,18–7,37% (during JIA treatment)	0.38 U/kg	Simultaneously	–
Case 2	Female	16 years	RF-positive polyarthritis	18	NSAIDs (Naproxen), methotrexate, sulphasalazine, adalimumab	10.2%– > 8.7%– > 7.2%– > 8.4%	0.94 U/kg	JIA diagnosed 12 years after T1D	–
Nagy et al. [11]	Female	15 years	Undifferentiated arthritis	More than 2	NSAIDs, methotrexate	14.2% (at diagnosis of JIA)	1.2 U/kg	JIA diagnosed 13 years after T1D	Autoimmune thyroiditis (diagnosed at the age of 11)
Olivieri et al. [12]	Female	11 years 2 months	RF-positive polyarthritis	13	NSAIDs (Naproxen), etanercept,	From 10.4% (at diagnosis of JIA) down to 7.8% (after implementation of etanercept)	0.8 U/kg (visible improvement since the start of biological therapy)	JIA diagnosed 10 years after T1D	Autoimmune thyroiditis (diagnosed at the age of 6 years 9 months)
Nolfi-Donegan et al. [13]	Male	11 years old	Systemic arthritis	More than 9	Prednisone, anakinra	n/d	N/D	JIA diagnosed 2 weeks after T1D	–
Mroczkowska-Juchkiewicz et al. [10]	Female	1 year 8 months	Oligoarthritis	4	Methotrexate, prednisone	14.45% (at diagnosis)	N/D	T1D diagnosed 2 months after JIA	Coeliac disease, autoimmune hepatitis

### Original case 1

An 18-month-old girl was admitted to the hospital because of low-grade fever, rhinitis, and cough for 2 days. She was transferred from the infectious diseases department to our endocrine unit because of hyperglycemia. During the week preceding the hospitalization, the girl manifested polyuria, polydipsia, 2 kg weight loss. On admission, she was lethargic and vomited. Hyperglycemia of 711 mg/dL (39.5 mmol/L) and ketoacidosis (pH = 7.31, base excess = − 16) were reported. Physical examination revealed the features of dehydration and Kussmaul breathing with acetone odor. She was diagnosed with type 1 diabetes. Initial treatment included intravenous insulin and rehydration, resulting in normoglycemia. Subcutaneous insulin therapy and a diabetic diet were implemented from the second day of hospitalization. Because of her young age, the decision was made to start a continuous subcutaneous insulin infusion (CSII) with a personal insulin pump system from the very beginning. Further laboratory investigation proved glycated hemoglobin HbA1c of 10.63%, low serum 25-hydroxyvitamin D concentration. According to the current recommendations, screening for autoimmune thyroid diseases and coeliac disease was carried out with a negative result [14]. After 5 days of insulin treatment, new symptoms appeared: swollen right knee (with limping), and after the next 9 days—swelling of the interphalangeal joints of the left hand. Treatment with an anti-inflammatory drug orally, topically, and intravenous antibiotic therapy (amoxicillin–clavulanate) was started. The musculoskeletal ultrasound of the affected articulations revealed joint effusion and synovial hypertrophy with concomitant tenosynovitis of flexors. Due to the spreading inflammatory process, a small dose of prednisone was used with a good response. Upon specialist advice by the rheumatologist, subsequent laboratory findings showed an increased erythrocyte sedimentation rate, lack of rheumatoid factor (RF), and anticitrullinated protein antibody (ACPA). After her parents were educated in insulin therapy and diabetes management, the patient was discharged home and referred to the rheumatologic follow-up. Two months following the initial hospitalization, an experienced rheumatologist made a definite JIA RF-negative diagnosis, according to ILAR criteria (second revision) [9]. The treatment with a small dose of oral GCS was maintained. Improvement was noted during the follow-up visit: she had normal physical activity, but walking difficulty with limited range of movement in the lower extremity persisted. The affected joints (right knee and proximal interphalangeal (PIP), distal interphalangeal (DIP), metacarpophalangeal (MCP) joints of the left II and III fingers) remained slightly swollen; however, the range of motion was full. Due to the number of involved joints, the treatment with intraarticular glucocorticoid injections was not implemented. A small dose of oral GCS (0.3 mg per kg of body weight of prednisone) was repeated, and oral methotrexate in the weekly dose of 10 mg/week was initiated, with further tapering and discontinuation of steroids. Disease remission was achieved in accordance with Wallace criteria [15]. Despite a few viral infections and the GCS use, the glycemic values were kept within an acceptable range (HbA1c of 7.1–7.32%). A continuous glucose monitoring system (CGM) was implemented to improve glycemic control.

The CGM consists of a sensor applied under the skin and measuring glucose level in the interstitial fluid, transmitter, and a display device (e.g., dedicated application on the smartphone) that continuously collects data from the sensor, enabling the patient to know their approximate current glycemia. After advanced educational training, parents were using CGM actively with a noticeable improvement of metabolic control of diabetes.

The above example shows that patients happen to be diagnosed with more than just diabetes from the very beginning, also at a very young age. Insulin therapy of such a child is a significant therapeutic challenge for both the medical team and caregivers and thus, requires close interactive cooperation.

### Original case 2

A 16-year-old girl with an 11-years-lasting history of T1D was admitted to the rheumatology unit because of chronic pain and swelling of multiple joints (knees, ankles, hips, shoulders, temporomandibular joints, of varying degrees of severity) having persisted for the previous two months. She had been treated with NSAIDs with no improvement. Based on the overall clinical picture, ultrasound examination, the results of laboratory findings (increased CRP, ESR), the presence of RF and ACPA, the diagnosis of polyarticular seropositive JIA was established according to ILAR criteria [9]. The treatment with oral methotrexate was started in an increasing weekly dose up to 15 mg/m^2^ (switched to subcutaneous administration due to poor tolerability of oral treatment). Due to the persistence of high disease activity, expressed as JADAS (Juvenile Arthritis Disease Activity Score) value of 14.8, sulphasalazine, another disease-modifying antirheumatic drug (DMARD), was added after 3 months [16]. Despite that 3-months-long combined therapy with two conventional DMARDs, response to treatment was insufficiently attained (JADAS 9.7); therefore, subcutaneous tumor necrosis factor-alpha (TNF-alpha) inhibitor (adalimumab) was started. During the initial phase of DMARD treatment, a small dose of oral glucocorticoids (0.5 mg/kg of body weight of prednisone) were used as ‘bridge’ therapy to achieve a quick alleviation while awaiting the full therapeutic effect of DMARDs. Afterward, due to JIA flares, oral GCS were continued temporarily. Her metabolic control of diabetes during the JIA treatment deteriorated much at first, resulting partly from poor compliance. The patient measured blood glucose rarely (3–4 times a day) and did not use the bolus calculator. Her blood glucose reached an average value of over 200 mg% (with a standard deviation over 100). She was covered by the assistance of a clinical board-certified psychologist (specialized in the management of chronic diseases) on the regular meeting sessions using individual psychotherapy. Psychological sessions were mainly aimed at strengthening acceptance of her chronic conditions, increasing her self-esteem, and motivating her to achieve the therapeutic goals gradually. She also met, on a regular basis, with specialized diabetes educators who taught her to respond appropriately to various scenarios in everyday life, to prevent hypo- and hyperglycemia. Along with intensive diabetes care, systematic reeducation, psychological support, and the use of a flash glucose monitoring system (FGM), stepwise amelioration in HbA1c from 10.23 to 7.2% was observed. The insulin requirements of this patient throughout her JIA treatment ranged from 0.92 to 0.94 U/kg. However, further exacerbations of the JIA and temporary use of oral GCS still worsened the diabetes control; the girl presented with HbA1c over 8%. In this patient, CSII combined with FGM were crucial. The implementation of the FGM system, 1 month after the patient had commenced adalimumab, allowed her to manage better the glycemic variability. Regular FGM use alleviated the complaints and laboratory indicators: the percentage of data collected from the sensor reached 87%, whereas the number of boluses per day increased from 6 to 9. The girl had to change insulin dosage every 2–3 days because of changing steroid treatment, adjusted to her rheumatologic disease. It remains a challenging situation for her because of persistent joint complaints and a decreased quality of life caused by blood glucose fluctuations. The combined treatment made it possible to achieve partial remission before she was transferred to an adult clinic (JADAS score of 4.5).

To summarize, insulin therapy in the adolescent patient was challenging, considering teenage non-compliance and reluctance to cope with the disease. In addition, the GCS therapy may have considerably constricted effective metabolic control of the patient. Furthermore, a significant clinical issue was an inadequate response to treatment of JIA, resulting in numerous modifications, including ultimately the initiation of biologic therapy.

## Discussion

T1D is the most common chronic metabolic disease of childhood [17]. The presence of one autoimmune process predisposes to the development of additional autoimmune diseases. Juvenile idiopathic arthritis, though being not the most common of them, is manifested by noncharacteristic clinical features, which may delay the diagnosis [18]. This is a common reason for misdiagnosis and may confer a risk of inappropriate treatment. T1D prevalence in patients with JIA reaches up to 0.5% [16], whereas JIA-prevalence in patients with type 1 diabetes (0.19%) is considerably greater than in the general population (0.05%) [19]. The coexistence of a few autoimmune diseases in one patient suggests that these conditions could have a common genetic background [5, 6]. The human leukocyte antigen (HLA), CTLA4, and PTPN22 as T-cell activation regulators are suspected of playing a key role in autoimmunity processes, i.e., the aberrant function of the molecules encoded by them may be responsible for the development of autoimmunity [20]. However, their role in JIA genetic susceptibility remains unclear [21].

Because JIA is a relatively rare disease, only a few cases of clustering JIA and type 1 diabetes were published in the last years [10–13]. The vast majority of available case reports were girls, typically for autoimmune diseases (except for T1D, where both sexes are at similar risk of the disease). In most reported patients, similarly to our cases, diabetes had been developed first, whereas juvenile rheumatoid arthritis was diagnosed afterward, sometimes followed by other autoimmune diseases [7]. Contrastingly, few studies showed that T1D developed after JIA, suggesting that T1D could have also developed because of GCS use or treatment with etanercept [7, 13, 22]. Significant problems of pediatric patients with T1D are maintaining glucose blood level stable via proper insulin dosage and managing with acute complications (hypo- and hyperglycemia). The issue is also to prevent the long-term sequelae [23–25].

Hyperglycemia and glucose variability is acknowledged as pro-inflammatory condition [26–28]. Therefore, it is crucial to avoid glycemic fluctuations in rheumatoid patients, not to stimulate and maintain inflammation, which may hinder the treatment of arthritis.

When considering the use of steroids in treating JIA in patients with concomitant T1D, one should be aware of that well-proven deleterious GCS effect on glucose metabolism [29, 30]. It means that GCS treatment can seriously harden treatment and lead to metabolic derangement. Another critical point is an increased requirement for exogenous insulin. Insulin resistance is increased by long-term steroid use and JIA as an inflammatory process. This creates complications at the metabolic level and with the insulin dosage and administration. Chronic use of excessive insulin doses could lead to weight gain, resulting in increased insulin resistance and disturbances in triglyceride and cholesterol levels, and dyslipidemia [31–33]. As for insulin administration, high insulin doses may also implicate local side effects like increased tissue traumatization because of a bigger volume of a single injection or bolus. This can be counteracted with modern concentrated insulin types U200, U300, U500. Unfortunately, it is challenging to find an appropriate insulin pump adapted to the above-concentrated insulin formulation. The broader application of biologic drugs during the previous years has greatly reduced the need for systemic GCS in children with JIA. According to the current guidelines, the use of GCS is restricted mainly to the management of extra-articular manifestation of systemic-onset JIA or particular indications such as macrophage activation syndrome (MAS), where GCS are used intravenously in high pulse doses [34]. In subtypes other than systemic-onset JIA, GCS should be used carefully because their potential adverse effects might prevail any benefits to articular disease. A short course of low-dose prednisone (up to 0.5 mg/kg/day) may be considered in patients with severe polyarthritis refractory to other drugs or as a bridging therapy while waiting for the therapeutic effect of a recently initiated conventional or biologic DMARDs) [35]. Intra-articular glucocorticosteroid (IAC) injections remain the mainstay of therapy of oligoarticular JIA, without systemic side-effects such as the impact on glucose metabolism. Moreover, multiple IAC injections may be considered in children with polyarticular JIA to induce prompt remission of synovitis while simultaneously initiating therapy with DMARDs and biologic agents [36]. This concept seems to be especially valuable in patients with concomitant diabetes regarding better glycemic control and lower risk of metabolic derangement; however, in presented two patients, due to the need for multiple arthrocentesis and no parental consent for this approach, the IAC injections were not implemented, which resulted in a need for the use of oral GCS and subsequent poorer diabetes control.

It is worth mentioning that some patients become GCS-dependent for proper JIA control; GCS have to be administrated due to recurrence of symptoms (or to be tapered slowly) [10, 13]. Notwithstanding, major therapeutic issues in JIA are not only side effects of GCS. Quality of life in JIA is deteriorated by morning stiffness, multifocal joint pain, contractures, and impaired mobility, which could result in disability in adulthood [37, 38].

Interestingly, biologic DMARDs administration in some JIA cases is associated with a significant improvement of HbA1c and better long-term metabolic control of diabetes. It should probably be recommended instead of GCS when it is possible [12, 39, 40]. Also, the patients receiving biologic DMARDs for JIA require less insulin therapy for diabetes [18]. Moreover, DMARDs therapy is suggested to prevent T1D development in patients with rheumatoid arthritis. Therefore, it could be an argument for starting biologic DMARD therapy early in the course of JIA management to potentially ameliorate the development of other autoimmune disorders [41, 42].

Another problem worth mentioning is collagen glycation. Patients with long-lasting type 1 diabetes have a very high joint stiffness prevalence (especially hand, shoulder, and lumbar) due to the collagen glycation process and accumulation of advanced glycation end-products [43]. Although these findings were observed in adults, an association between joint stiffness and increased glycated hemoglobin might be unnoticed at a younger age. A speculative question arises whether the onset of such problems may occur already in childhood and adolescence (with a sufficiently long duration of diabetes and poor metabolic control) and become a kind of a “mask” of the morning stiffness symptom which has been often attributed to rheumatologic disease including JIA).

The psychological and psychosocial aspects of JIA and T1D as overlapping conditions and their impact on a pediatric patient are also important [44, 45]. It is extremely difficult for a young person to cope with one chronic disease, let alone two or more. Psychological support can improve diabetes metabolic control and, consequently, affect the patient's well-being and quality of life in a long-term perspective [46]. For this reason, the clinical psychologist should be included in the interdisciplinary team taking care of such a patient. Another questionable issue remains the optimal pattern for the multidisciplinary service encompassing pediatric rheumatology, diabetes care, and transition schedule between pediatric and adult care.

It is worth mentioning that our study remains limited by a few issues. First, conclusions from our research regarding children cannot be generalized to the adult population. Second, the follow-up of our patients is considerably short (it differs in cited cases), which may bias our picture of the illness in the longer term. The following limitation is the retrospective character of the study. Because our information has been gathered from electronic medical records, our findings are limited by the possibility of gaps in the documentation that cannot be avoided despite careful data gathering.

To conclude, the treatment of a multimorbid patient requires the cooperation of several trained specialists. Diabetologists should be aware of other autoimmune diseases, including rheumatological conditions, and thus be alert to specific clinical features, e.g., joint stiffness or pain in their patients. T1D coexistence with other autoimmune conditions may deteriorate metabolic control and may considerably impede insulin treatment, especially during systemic GCS use, even despite its small dose. Hyperglycemia can worsen JIA therapy by induction and maintaining inflammation. Personal insulin pumps and CGM systems help adjust insulin dosing and maintain metabolic control during active inflammation and GCS therapy. Moreover, the broader use of IAC injections (even in polyarthritis) and earlier treatment with modern biologic drugs may reduce the need for systemic GCS. In the future, hybrid and fully closed-loop insulin pump systems may facilitate the therapy even more.

## Data Availability

The raw data supporting the conclusions of this article will be made available by the authors, without undue reservation, the medical history of the patients.

## Abbreviations

T1D: Type 1 diabetes mellitus
JIA: Juvenile idiopathic arthritis
GCS: Glucocorticosteroids
DMARD: Disease-modifying antirheumatic drug
ISPAD: International Society for Pediatric and Adolescent Diabetes
ILAR: International League of Associations for Rheumatology
CSII: Continuous subcutaneous insulin infusion
CGM: Continuous glucose monitoring system
PIP: Proximal interphalangeal joint
DIP: Distal interphalangeal joint
MCP: Metacarpophalangeal joint
ESR: Erythrocyte sedimentation rate
JADAS: Juvenile Arthritis Disease Activity Score
RF: Rheumatoid factor
ACPA: Anti-citrullinated peptide antibody
TNF-alpha: Tumor necrosis factor-alpha
FGM: Flash glucose monitoring system
HLA: Human leukocyte antigen
